# The effects of antiviral treatment on breast cancer cell line

**DOI:** 10.1186/s13027-017-0128-7

**Published:** 2017-03-23

**Authors:** Madina Shaimerdenova, Orynbassar Karapina, Damel Mektepbayeva, Kenneth Alibek, Dana Akilbekova

**Affiliations:** 1 National Laboratory Astana, Nazarbayev University, Qabanbay Batyr Avenue 53, Astana, 010000 Kazakhstan; 2 Nazarbayev University Research and Innovation System, Nazarbayev University, Astana, Kazakhstan; 3 Locus Solutions LLC, Solon, OH USA

**Keywords:** MCF7 breast cancer cell line, Acyclovir, Antiproliferative effect, ALDH activity

## Abstract

**Background:**

Recent studies have revealed the positive antiproliferative and cytotoxic effects of antiviral agents in cancer treatment. The real effect of adjuvant antiviral therapy is still controversial due to the lack of studies in biochemical mechanisms. Here, we studied the effect of the antiviral agent acyclovir on morphometric and migratory features of the MCF7 breast cancer cell line. Molecular levels of various proteins have also been examined.

**Methods:**

To evaluate and assess the effect of antiviral treatment on morphometric, migratory and other cellular characteristics of MCF7 breast cancer cells, the following experiments were performed: (i) MTT assay to measure the viability of MCF7 cells; (ii) Colony formation ability by soft agar assay; (iii) Morphometric characterization by immunofluorescent analysis using confocal microscopy; (iv) wound healing and transwell membrane assays to evaluate migration and invasion capacity of the cells; (v) ELISA colorimetric assays to assess expression levels of caspase-3, E-cadherin and enzymatic activity of aldehyde dehydrogenase (ALDH).

**Results:**

We demonstrate the suppressive effect of acyclovir on breast cancer cells. Acyclovir treatment decreases the growth and the proliferation rate of cells and correlates with the upregulated levels of apoptosis associated cytokine Caspase-3. Moreover, acyclovir inhibits colony formation ability and cell invasion capacity of the cancer cells while enhancing the expression of E-cadherin protein in MCF7 cells. Breast cancer cells are characterized by high ALDH activity and associated with upregulated proliferation and invasion. According to this study, acyclovir downregulates ALDH activity in MCF7 cells.

**Conclusions:**

These results are encouraging and demonstrate the possibility of partial suppression of cancer cell proliferation using an antiviral agent. Acyclovir antiviral agents have a great potential as an adjuvant therapy in the cancer treatment. However, more research is necessary to identify relevant biochemical mechanisms by which acyclovir induces a potent anti-cancer effect.

**Electronic supplementary material:**

The online version of this article (doi:10.1186/s13027-017-0128-7) contains supplementary material, which is available to authorized users.

## Background

Current cancer therapy includes the use of chemotherapeutic agents, surgery and radiation therapy. It is estimated that four types of viruses alone could cause 12% of cancer cases worldwide. These are human papillomavirus (HPV), hepatitis B (HBV), hepatitis C (HCV), and Epstein–Barr virus (EBV) [1]. Investigation of the virus-associated cancer serves as a unique platform for the development of novel strategies to prevent the development of infection that can predispose tumorigenesis. Studies on antiviral drug treatments demonstrate promising results on the prognosis through the prevention of carcinogenesis. Administration of the antiviral agents in combination with the anticancer drugs is known for positively influencing the effectiveness of the treatment [2]. This combined therapy is termed as an adjuvant antiviral therapy [3]. Complex combinations of the chemotherapeutic agents together with the antiviral drugs are used to treat a number of infection-associated malignancies such as Kaposi sarcoma, hepatocellular carcinoma (HCC) and nasopharyngeal carcinoma [4, 5]. A study based on the electronic health records of 2671 adult participants diagnosed with chronic HBV infection from 1992 to 2011 also indicates that an antiviral treatment against chronic HBV infection markedly decreases the incidence of HCC in the treated patients [5].

Adjuvant antiviral therapy also has a reported antiproliferative effect in some types of cancer [2]. Treatment of breast cancer cells with ribavirin decreases the level of one of the biomarkers of this malignancy, eukaryotic translation initiation factor (eIF4E), which is usually elevated in more than 25% of cancer cases [6]. Ribavirin disrupts the structure of eIF4E, leading to the inhibition of cyclin D1 and expression of NBS1 oncogene [7, 8].

Namba et al. demonstrated the use of zidovudine, an antiviral drug, in combination with gemcitabine, a chemotherapeutic agent - in an attempt to overcome a gemcitabine resistance for the pancreatic cancer treatment. In this type of malignancy, the gemcitabine resistance is associated with a decreased level of human equilibrative nucleoside transporter 1 (hENT1) and acquisition of epithelial-to-mesenchymal transition (EMT) - like phenotype. The zidovudine adjunct therapy was shown to reverse both events in this study [9]. Furthermore, authors demonstrated that activation of Akt-GSK3β-Snail mechanism, one of the major signaling pathways during gemcitabine resistance, is inhibited by zidovudine so that gemcitabine-resistant cancer cells were resensitized.

Although there is a plethora of evidence suggesting the beneficial effect of the antiviral agents in cancer treatment, the therapeutic benefit of their use in cancer treatment remains a grey area due to the lack of studies of the biochemical mechanisms. Antiviral agents such as acyclovir and ribavirin have been reported to have a suppressive effect on the proliferation and ability to increase an apoptosis in various cancers [7, 8]. Acyclovir was discovered 40 years ago and remains one of the main existing therapies for herpes simplex virus (HSV) infections. This drug is a highly potent inhibitor of this virus and commonly used for the treatment of the infections caused by the herpes viruses, CMV and EBV. It also has a low toxicity for the normal cells [10].

In the present study, we propose to investigate how cancer cells respond to the antiviral agent as acyclovir in vitro and whether this treatment can affect the metastatic phenotype of cancer cells. We report results on the potential effect of acyclovir treatment on the cell proliferation, invasion capacity, cytotoxicity, and the expression of tumor suppressing genes.

## Methods

### Cell culture

Breast cancer cell line MCF7 (American Type Cell Collection, ATCC® HTB 22™) and human breast epithelial primary cells (Celprogen, Benelux, Netherlands) were cultured in a complete media (CM) (Dulbecco’s modified Eagle’s medium (DMEM) (D6421, Sigma-Aldrich, St Louis, MO, USA) supplemented with 10% fetal bovine serum (12103C-500 ml, Sigma-Aldrich, Buchs, Switzerland), 100 U/mL penicillin, 100 μg/mL streptomycin and 25 ug/mL Amphotericin B (SV30079.01, HyClone, Thermo Scientific, South Logan, Utah, USA) at 37 °C in 5% CO_2_. Cells were subcultured every three days using 0.25% trypsin-EDTA (25-052-CI, Cellgro, Mediatech Inc, Manassas, VA, USA) for detachment.

### Treatment with Acyclovir

Antiviral agent – acyclovir (ACV) in powder form was purchased from Sigma-Aldrich (PHR1254-1G, St Louis, MO, USA). A 10 mM (stock) solution was prepared in phosphate buffered saline (PBS) and sterilized through filtering (0.45 μM PVDF 25 mm filters, (094.01.003, Isolab, Wertheim, Germany). Stock solution was stored at -20 °C. MCF7 cells were cultured in 12-well plate at 26,000 cells/cm^2^ (92412, TPP, Switzerland) in the presence of 5 uM acyclovir solution and incubated for 72 h at 37 °C in 5% CO_2_. In a positive control experiment, cells were cultured in the absence of acyclovir. A control of acyclovir without cells was also conducted. All experiments were performed in triplicate.

### Viability assay

Cell viability after the acyclovir treatment was evaluated with MTT (3-(4,5-dimethylthiazol-2- yl)-2,5-diphenyltetrazolium bromide (M5655-1G, Sigma Aldrich, St Louis, MO, USA) assay. Concentration of 5 mg/ml was achieved by reconstituting MTT in DI water. After removal of the supernatant from the wells, 500 μl of warm culture media and 50 μl of MTT solution were added in each well for two hours at 37 °C in 5% CO_2_. Then 400 μl of media was removed and crystals of formazan were diluted with 500 μl of dimethyl sulfoxide (DMSO) (D4540-100 ml, Sigma Aldrich, St Louis, MO, USA). Absorbance of cells was measured at 570 nm (BioTek *ELx*800 plate reader, Winooski, Vermont, USA).

### Proliferation assay

Proliferation in MCF7 cells was determined by plating 6500 cells/cm^2^ into 12-well plate and cultured in a medium with and without ACV. After overnight incubation cells were detached with trypsin and counted using automated cell counter (TC20™, 1450102, Bio-rad Laboratories, Berkeley, California) at 24, 48, 72 and 96 h.

### Soft agar assay

The ability of cancer cells to form colonies was characterized using a soft agar assay. This assay required 21 days of growth on the soft agar medium. At the end of 3 weeks, a number of colonies formed per petri dish were counted using a crystal violet stain. Briefly, 1% sterile agar solution was warmed in a microwave and place to 37 °C water bath to cool down. 500 μg of agarose powder (BP165-25, Thermo Fisher Scientific, Fair Lawn, New Jersey, USA) was dissolved in 50 mL distilled water. The bottom of the petri dish (502014-07P, Sterilin petri dishes 9.6 cm^2^, Dynalon Labware, Rochester, NY, USA) was coated with 0.7% agar and 0.3% CM by adding 3 ml/dish at room temperature for 30 min. following this, the upper layer of 3 mL of agar solution with 0.3% agar and 0.7% cell suspension (3125 cells/cm^2^) was plated. The top agar layer was allowed to solidify and then incubated for 3 weeks at 37 °C in 5% CO_2_. CM was refreshed 2 times a week. In 21 days crystal violet was used as a staining for colonies (C3886, Sigma-Aldrich, Munich, Germany) and counting was performed using Leica DMI3000 B light microscope.

### Cell staining for fluorescent imaging

#### Morphological analysis

Glass coverslips were cleaned for 2 h in 200 mL ethanol, 50 g NaOH, and 300 mL DI water and finally rinsed with PBS. Cells were seeded on glass coverslips at 10,500 cells/cm^2^, placed in petri dishes (Sterilin petri dishes 9.6 cm^2^) and incubated at 37 °C in 5% CO_2_ overnight before the treatment. After the 72 h incubation with acyclovir, coverslips with cells were rinsed briefly with PBS for thrice for 5 min each time. 4% paraformaldehyde was used as a fixative for 10 min at room temperature. The samples were blocked with 1% BSA and 0.3% Tween-20 (P2287-500 ml, Sigma-Aldrich, St Louis, MO, USA) in PBS and incubated at room temperature for 1 h. Following 1 h incubation, α-tubulin rabbit mAb Alexa Fluor® 488 conjugate (322588, Invitrogen, Life Technologies, Rockford, IL, USA) diluted as 1:200 was used for staining and incubation at 4 °C overnight in the dark. After 24 h, the coverslips were washed 3 times for 5 min with PBS and then incubated with 0.1 μg/mL DAPI for 2 min. After rinsing again with PBS, aqueous mounting medium (ab128982, Abcam, Cambridge, MA) was used for mounting coverslips on microscope slides. Finally, coverslips were sealed with a clear nail polish. Images were acquired using EVOS® FLoid® Cell Imaging Station (4471136, Life Technologies, Carlsbad, California, USA). Cellprofiler software was used to evaluate morphometric features of treated MCF7 cells (Broad Institute, www.cellprofiler.org). The equation used for the quantitative measurement of the shape of the cell is given below. This equation uses form factor, FF:

1 $$ \mathrm{F}\mathrm{F}=4\ast \uppi \ast \mathrm{area}/\mathrm{perimete}{\mathrm{r}}^2 $$

The pipeline for this analysis included four modules: Identify Primary Objects, Identify Secondary Objects, Identify Tertiary Objects and Measure Object Size Shape [11]. Area ratio was calculated by dividing area of nucleus over area of cytoplasm.

#### Immunofluorescence staining for the presence of E-cadherin

4% paraformaldehyde in PBS was used as a fixative for 10 min at room temperature and 0.1% Triton was used as a permeabilization solution for 10 min on ice. Following this, cells were washed 3 times with ice cold PBS and blocked with 1% BSA in PBST at room temperature for 1 h. Blocked cells were stained with 1 mg/ml of Ms mAb to E-cadherin (ab1416, Abcam, Cambridge, MA, USA) in 1% BSA in PBST in a humidified chamber for 24 h at 4 °C. The stained samples were then washed 3x5 min with PBS and incubated overnight with a 2 mg/ml of secondary antibody goat pAb to Ms IgG Alexa fluor 488 (ab150113, Abcam, Cambridge, MA, USA) in 1% BSA in PBST. Coverslips were washed 3x5 min with PBS in the dark and incubated with DAPI for 2 min, and mounted on the microscope slides using glycerol. Images were acquired using EVOS® FLoid® Cell Imaging Station (4471136, Life Technologies, Carlsbad, California, USA).

### Aldehyde dehydrogenase activity colorimetric assay (ALDH)

NAD-dependent ALDH activity was measured using colorimetric assay kit (MAK082-1KT, Sigma Aldrich, Saint Louis, MO, USA) and performed as described in the manufacturer’s instructions. The absorbance was measured at 450 nm on BioTek *ELx*800 plate reader. Measurements were recorded every 3 min until the value of the control sample exceeded the value of the most active standard (10 nmole/well). The following equation was used to calculate the activity of the enzyme:

2 $$ \frac{\mathrm{ALDH}\ \mathrm{Activity} = \mathrm{NADH}\ \mathrm{Amount}\ \left(\mathrm{nmole}\right)\ \mathrm{x}\ \mathrm{Sample}\ \mathrm{Dilution}\ \mathrm{Factor}}{\left(\mathrm{Reaction}\ \mathrm{time}\right)\ \mathrm{x}\ \mathrm{Sample}\ \mathrm{volume}\ \left(\mathrm{mL}\right)} $$

### Transwell migration assay

Cells were cultured for 72 h with and without acyclovir at 37 °C in 5% CO2, and then in serum-free medium for another 24 h at 37 °C in 5% CO_2_. After detachment with 0.05% trypsin- EDTA the cells were re-suspended in a serum-free medium. Upper insert was filled with 100 μl of the cell suspension (~9x10^4^-1x10^6^ cells per well) while reservoir chamber was filled with 600 μl of culture medium. Migration of cells was monitored at 3, 6, and 12 h at 37 °C in 5% CO_2_. Crystal violet was used as the staining solution to distinguish between migrated and non-migrated cells. A cotton swab was used to remove the cells that were left in the upper chamber of the membrane. Those cells that migrated through the insert were examined and counted with bright-field microscope (LEICA DMI3000 B, Wetzlar, Germany).

### Wound healing

Cell culture was performed at a concentration of 260,000 cells/cm^2^ in 12-well plates incubated overnight at 37 °C in 5% CO_2_. The medium was then substituted with CO_2_ free media with and without ACV. A scratch using sharpened toothpick was made on the surface of the well to simulate a wound in vitro. Four randomly areas in a well were selected and imaged in 10 min intervals for 12 h using time-lapse microscopy system (AMAFD1000, EVOS FL Auto imaging system, Life Technologies, Thermo Fisher Scientific, Carlsbad, CA, USA). The images obtained were processed using ImageJ (image processing software). The sequence of images was analyzed and the open wound area was measured for each image at every hour. A scatter plot of wound area measurements (units) vs. time (in hours) was generated as seen in Additional data 5. A line-of-best fit was used to calculate the slope, which corresponded to the rate of migrated cells.

### E-cadherin, C-Myc, NF-kB p65 and caspase-3 levels colorimetric assays

Secretion levels of E-cadherin in a cell culture were measured using a human E-cadherin ELISA colorimetric assay kit (99-1700, Invitrogen, Novex by Life Technologies, Frederick, MD, USA) and performed as described in the manufacturer’s instructions. Cellular levels of C-Myc, NF-kB p65 and caspase-3 in cell lysates were measured using a human C-Myc ELISA colorimetric assay kit (KHO 2041, Novex by Life Technologies, Frederick, MD, USA); a human NF-kB p65 Total ELISA colorimetric assay kit (KHO 0371, Novex by Life Technologies, Frederick, MD, USA) and a human caspase-3 ELISA Kit (KHO 1091, Novex by Life Technologies, Frederick, MD, USA).

Cell lysates were prepared using a 1 mM phenylmethylsulfonyl fluoride (PMSF) cell extraction buffer, a protease inhibitor cocktail (78439, Thermo Scientific, Rockford, IL, USA) and RIPA buffer (89900, Thermo Scientific, Rockford, IL, USA). Lysis was performed by adding 500 μl extraction buffer to the cell pellet for 30 min on ice while vortexing every 10 min. Then the cells were placed in the microcentrifuge tubes at 13,000 rpm for 10 min at 4 °C. Lysates were stored at -80 °C. All experiments were performed in triplicate. Results were expressed as mean concentration ± standard deviation and normalized to the number of live cells.

### Apoptosis assay

Programmed cell death was studied using annexin V-FITC apoptosis detection kit (331200, Invitrogen, Camarillo, CA, USA). Treated and control cells were harvested and re-suspended in CM to obtain a target concentration of 1×10^6^/ml in 1.5 ml Eppendorf tubes. Cells were centrifuged for 1 min at 3000 rpm, washed with ice cold PBS and centrifuged again. Cell pellets were re-suspended in 190 ul of 1x binding buffer with 10 ul Annexin V-FITC and 10 ul of 20ug/ml propidium iodide for 15 min at room temperature in the dark. Apoptosis of the cells was analyzed by flow cytometry (SORP FACSAria -II with 6 lasers, BD Biosciences, San Jose, USA).

### Statistics

All reported results below are presented as mean values ± standard error values. To calculate differences between means, one-way analysis of variance (ANOVA) was implemented, where a null hypothesis was accepted when all means were equal. Population differences were calculated only for a treatment and a control within the cell line. If at least one mean was different, a follow-up Tukey’s HSD test to compare between groups and calculate p-values of each sample (α = 0.05).

## Results

Viability of MCF7 cells and breast epithelial cells after the treatment with 5 μM ACV were measured and normalized to the untreated culture cells [Additional file 1]. MCF7 cells and breast epithelial cells demonstrated 80,687 and 97,194% of viable cells after the ACV treatment, respectively.

**Fig. 1:**
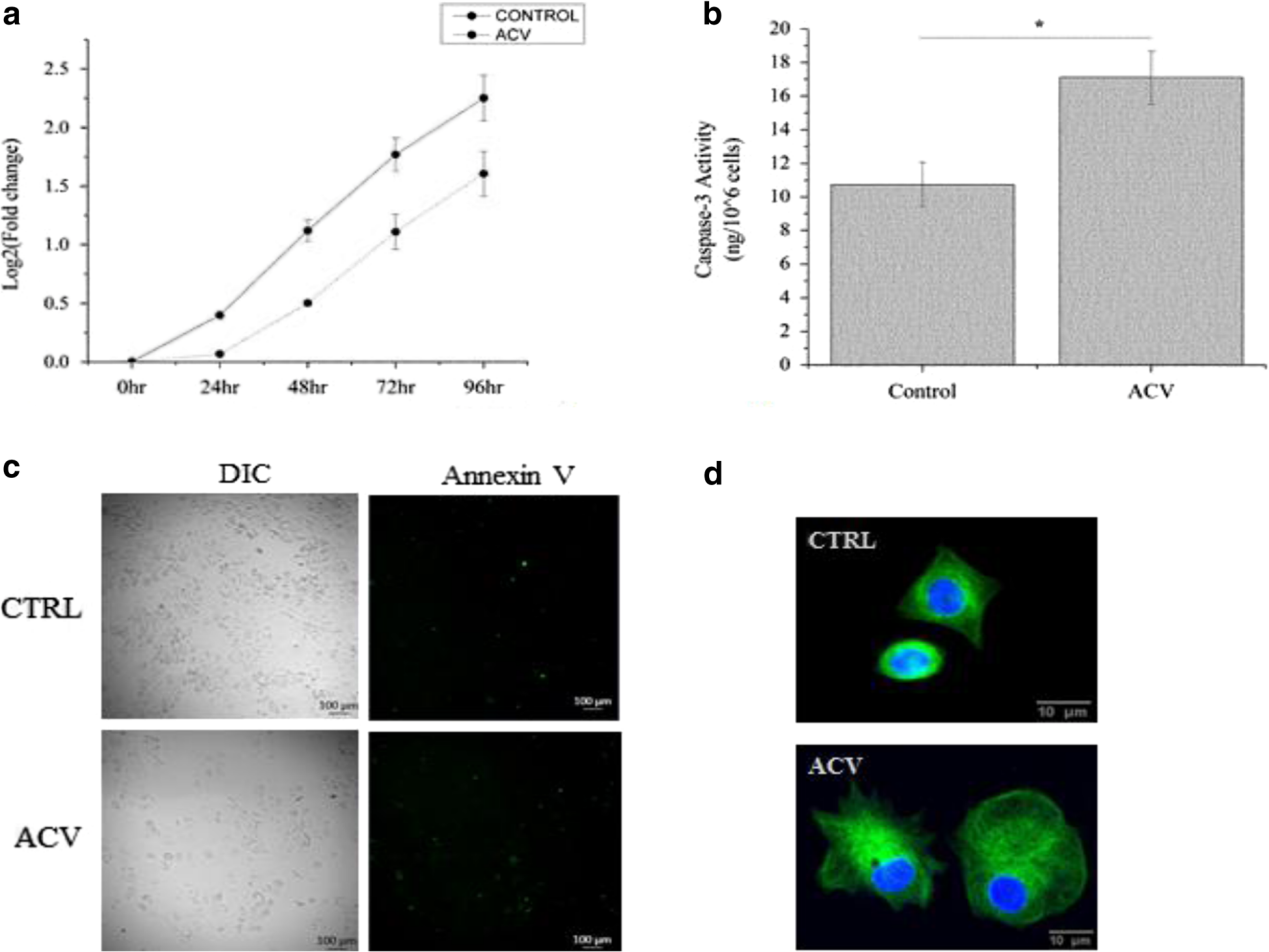
The effect of ACV treatment on proliferation and morphometric features of MCF7 cells. **a** Relative cell number of MCF7 cells proliferation. Cells were counted at 0, 24, 48 and 72 h post treatment. **b** Caspase-3 activity (ng/10^6 cells) in MCF7 cells treated with ACV. **c** Annexin V staining of apoptotic MCF7 cells. Left panel is bright field images; right panel is Annexin V staining images. *Green* is cells stained with FITC Annexin V. Magnification 10X on Microscope Cell Observer SD Carl Zeiss with CMOS ORCA-Flash 4.0 V2. **d** Nuclei and cytoskeleton staining of MCF7 cells. *Blue* is nuclei stained by DAPI; *green* is cytoskeleton stained with anti- alpha tubulin antibody. Magnification 20X on Microscope Cell Observer SD Carl Zeiss with CMOS ORCA-Flash 4.0 V2. For better visualization color enhancement was applied using ZEN software (for current images only)

First, we sought to evaluate the influence of ACV on the proliferation ability and apoptosis of MCF7 cells. Regulated interplay between apoptosis and cell proliferation is essential for the processes like tissue development and deregulation mechanisms [12]. Deregulated mechanism of apoptosis that correlates with an uncontrolled cell proliferation eventually leads to the carcinogenesis [13]. Several antiviral agents demonstrated the ability to inhibit proliferation and increase proapoptotic activity of the cancer cells [2]. Here, the rate of proliferation decreased during 96 h treatment with ACV (Fig. 1a). ACV significantly increased a population doubling time compared to the control cells ∾1.4 fold [Additional file 2]. Caspase-3 level in MCF7 cells was estimated to evaluate the apoptotic activity of cancer cells in response to ACV treatment. ACV upregulated caspase-3 expression in the treated cells ∾1.7 compared to the control cells (*p* < 0.05) (Fig. 1b). Annexin V staining and flow cytometry analysis demonstrated a slight increase of the number of the apoptotic cells in response to ACV treatment (*p* > 0.05 for the late and early apoptosis) (Fig. 1c, Additional file 3).

When examining normal cells and cancerous cells under the microscope, we observed distinctive external characteristic features. Results of the IF staining indicate that cancer cells underwent changes in their morphological characteristics in response to ACV treatment (Fig. 1d). FF shape descriptor was used quantitative characterization of these changes, where FF value of 1 served as a detector of a circular shape and 0 indicated linear or star shaped object [Additional file 4]. ACV treated cancer cells displayed a decrease of FF compared to the control cells from 0.828 ± 0.014 to 0.659 ± 0.012, indicating that ACV treated cells were more spread out with a non-uniform shape (∾1.25 fold). Furthermore, ACV treated cancer cells had a larger cytoplasmic volume compared to the control cells.

**Fig. 2:**
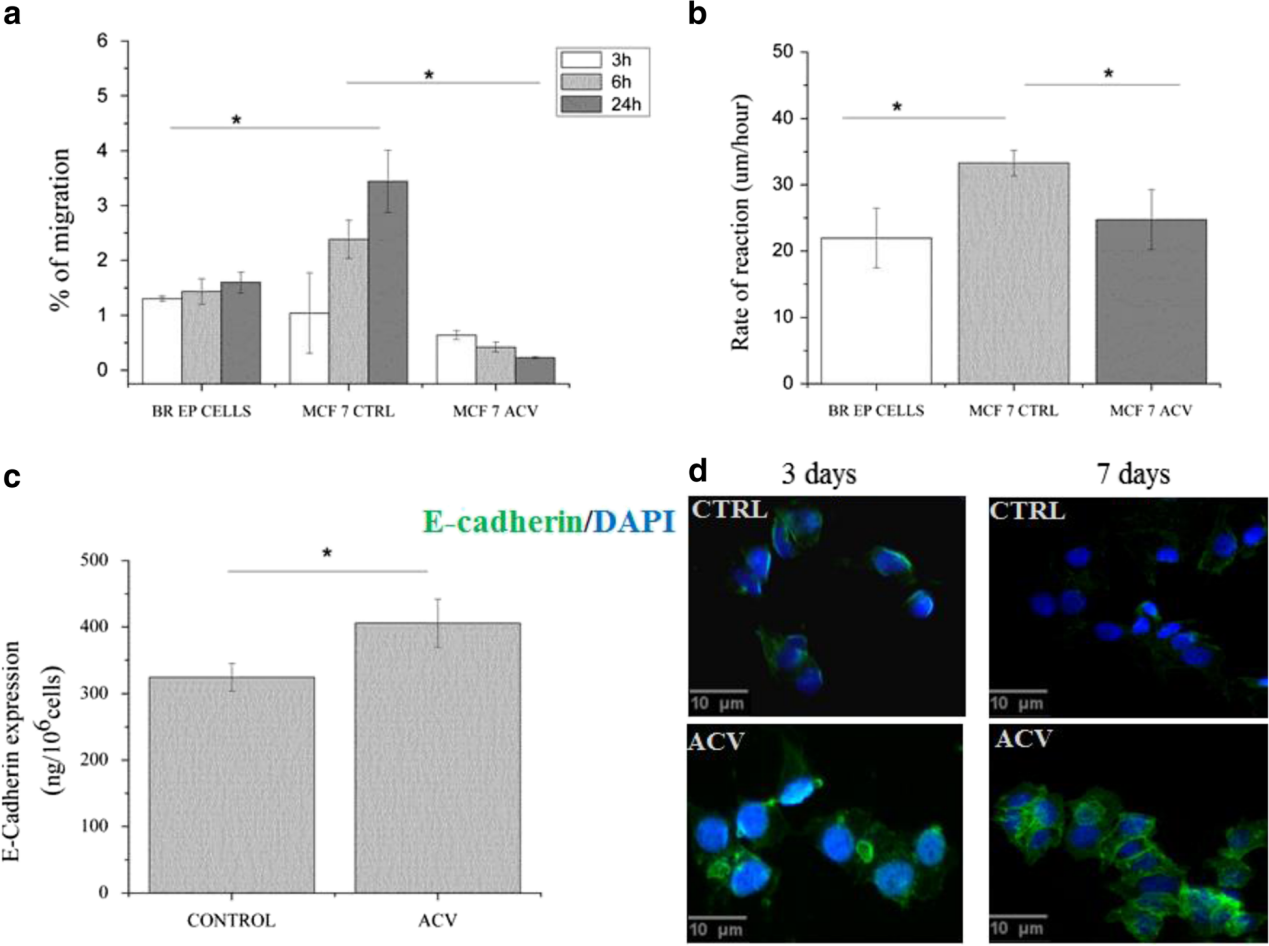
Migratory characteristics and E-cadherin expression of MCF7 cells in response to ACV treatment. **a** Migration of breast epithelial and MCF7 breast cancer cells through Transwell membrane. Migration percentage was counted at 3, 6 and 24 h post-seeding. **b** Rate of reaction of wound healing of breast epithelial cells and MCF7 breast cancer cells. **c** E-cadherin expression (ng/10^6 cells) in MCF7 cells treated with ACV. **d** Immunofluorescence staining of protein level of E-cadherin (*green*) in MCF7 cells after 3 and 7 days treatment. Nuclei are shown in *blue*. Error bars represent a 95% confidence interval based on the standard deviation. (*) indicates *p* <0.05 as compared with other samples and for pairwise comparison. One way ANOVA tests followed by Tukey’s test were used for statistical analysis. The data for each cell type were obtained from the same culture experiment

The effect of ACV treatment on the migratory and invasive capacities of the breast cancer cells was also tested. Various environmental factors can modulate the motility of cancer cells and affect invasion capacity of these cells. Teng et al. showed that antiviral drug ribavirin causes a considerable suppression of the migration of renal cell carcinoma cell lines [14]. Boyden chamber migration assay was performed to assess whether ACV affects MCF7 chemopolarised migration. As seen in Fig. 2a, ACV treatment reduces the number of cells migrating towards the chemoattractants as compared to the control cells. The cell invasion capacity of the treated cancer cells dropped ∾15 times compared to the untreated cells (*p* < 0.05). Fig. 2b shows the effect of ACV on the collective motility and rates of migration of both normal and cancer cells. The rate of the wound closure of ACV treated cancer cells decreased significantly compared to the control cells with ∾1.34 fold and was comparable to the rate of normal breast epithelial cells (24.74 μm/h and 21.95 μm/h for ACV treated and normal cells, respectively) (*p* < 0.05) [Additional file 5].

E-cadherin is secreted in most of the epithelial tissues and normal expression of E-cadherin has been reported to inhibit metastasis and invasion by suppressing epithelial-mesenchymal transition as well as stimulating cell-cell adhesions [15, 16]. We observed a significant increase of E-cadherin secretion in ACV treated cells compared to the control ∾1,25 (*p* < 0.05) as shown in Fig. 2c, d.

**Fig. 3:**
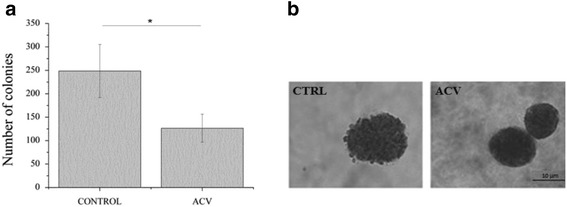
ACV altered ability to form colonies of MCF7 breast cancer cells (**a**) Number of colonies of MCF7 cells in response to ACV treatment. **b** Representative images of colonies formed. Duration of growing on a soft agar was 21 days. Error bars represent a 95% confidence interval based on the standard deviation. (*) indicates *p* <0.05 compared with control and other samples. One way ANOVA followed by Tukey’s test were used for statistical analysis. The data for each cell type were taken from the same culture experiment

Next, we evaluated the action of ACV on the ability of MCF7 cells to form colonies. This distinguishing feature of the cell transformation and its deregulated growth served as a marker to distinguish between cancer and normal cells, since normal cells do not have the ability to grow in semisolid matrices [17]. ACV treatment effectively decreased (∾ 2 fold) the number of colonies formed by cancer cells compared to the untreated cells as seen in Fig. 3a. Also the differences in external features are clearly observable in rough scabrous surface of control cells (R - shape) versus smoother surface of ACV treated cells (S -shape). (Fig. 3b).

**Fig. 4:**
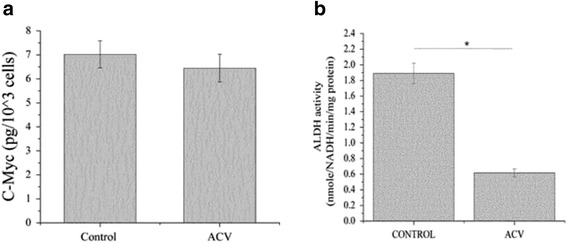
**a** C-Myc (pg/10^3 cells) and (**b**) ALDH activity (nmol NADH/min/mg protein) expressions of MCF7 cells in response to ACV treatment. Error bars represent 95% confidence interval based on the standard deviation. (*) indicate *p* <0.05 as compared with other samples and for pairwise comparison. One way ANOVA tests followed by Tukey’s test were used for statistical analysis. The data for each cell type were obtained from the same culture experiment

The concentration changes of C-Myc protein secreted by MCF7 cells were determined in response to ACV treatment. C-Myc oncogene regulates cellular growth and metabolic mechanisms as well as their interconnection [18]. Our results demonstrate a non-significant effect of ACV on the level of secretion of C-Myc in MCF7 cells. The concentration of C-Myc protein was similar to the control (Fig. 4a). Additional examinations of the viral protein NF-kB p65 demonstrated an elevated level after treatment with ACV [Additional file 6].

ALDH activity is one of the detectors of cancer progression [19]. Upregulated expression of ALDH1, one of the isoforms of ALDH family, has been reported as a crucial event in the breast cancer prognosis correlated with a poor clinical outcome [20, 21]. Moreover, studies show that ALDH activity is linked to the differentiation and expansion. It is also associated with a self-protective ability [22]. Our assessment of the effect of ACV treatment on ALDH activity of breast cancer cells shows a significant decrease (~3 fold) of ALDH activity in MCF7 cells compared to the control cells (Fig. 4b).

## Discussion

Recently, several antiviral agents have been found to possess the ability to decrease the rate of the cells’ proliferation and to promote apoptosis in cancer cells. However, despite the compelling results supporting the clinical use of antiviral agents as an adjuvant therapy in cancer treatment, there is still a lack of studies of the biochemical mechanisms of their anticancer effects [23, 24]. There are several factors that might be involved in a therapeutic approach of the antiviral adjuvant therapy in cancer treatment: an antiviral agent selectively targets cancer related viruses or post-chemotherapy infections and may also result in the cytotoxic and antiproliferative effects on the cancer cells, causing apoptosis [2]. Our implementation of ACV as an antiviral strategy demonstrated a positive effect on the prevention as well as successful predictive capability in the treatment of various types of malignancies.

In this study, we used acyclovir (ACV) to examine the potential of the antiviral treatment on MCF7 breast cancer cell line. Acyclovir is an antiviral drug used in treating infections of *Herpesviridae* family [25]. In several studies antiviral agents similar to ACV were used as an adjuvant therapy along with the chemotherapy [26, 27]. Records of the patients diagnosed with nasopharyngeal carcinoma demonstrate a suppression of the tumor growth for several months where injection of antiviral was used in tandem with the chemotherapy [26]. Moreover, *in situ* hybridization shows that the tumor cell populations were reduced in EBV-encoded RNAs [26]. In another study, the effect of acyclic nucleoside phosphonate against HPV-associated cancer was examined. The results indicate that the adjunct therapy using a cytotoxic drug and acyclic nucleoside phosphonates is more effective than one therapy alone. The authors also report an inhibited rate of the virus replication that led to a decreased expression of the viral oncoproteins and upregulation of the tumor-suppressor genes [27].

Based on our results, we conclude that ACV as an antiviral agent has a potential suppressive effect on MCF7 breast cancer cells. ACV does not affect viability of non-cancerous breast epithelial cells, while showing a decrease of the viability of MCF7 breast cancer cells. Observed morphological changes and apoptosis analysis demonstrated the ability of ACV to affect the process of programmed cell death of MCF7 cells. The mechanism of apoptosis requires a number of proteins that regulate a proper cell death. One of these proteins is caspase-3 which is included in a family of cysteine proteases [28]. An upregulated level of the apoptosis associated cytokine Caspase-3 was detected in ACV treated cells, correlating with the higher number of apoptotic cells and decreased rate of the cancer cell proliferation. Previously, it was reported that zidovudine treatment combined with a chemotherapeutic agent cisplatin has increased the apoptosis level of head and neck cancer cells [29]. This synergistic strategy of zidovudine and cisplatin was shown to trigger abnormal regulation of the mitochondria, increase of oxidative stress response and cause a significant cytotoxic effect on the cancer cells through the inhibition of a thiol metabolism [29]. Quantitative analysis revealed a moderate effect of acyclovir with a slight increase of the apoptotic cells.

ACV was also able to decrease the rate of the growth, colony formation ability, and cell invasion capacity of MCF7 breast cancer cells. These observations correlate with an upregulated secretion of E-cadherin in ACV treated cells. As previously mentioned, E-cadherin is an essential marker in the building of cell-to-cell adhesion and downregulation of this protein leads to the stimulation of invasion and metastasis [30].

Previous studies also report that antiviral agents can affect the secretion of the specific translation initiation factors, oncogenes, and angiogenic genes [8, 31, 32]. Borden et al. showed the ability of ribavirin to decrease the oncogenic potential of eukaryotic translation initiation factor (eIF4E) in the case of acute myeloid leukemia with a poor prognosis. Ribavirin binds eIF4E around the m7G cap-binding site leading to the reduction of affinity of this translation initiator factor [32]. While, we observed that ACV did not affect the expression levels of C-Myc oncogene, suggesting that antiviral agents might have a selective impact on the secretion of specific proteins.

Moreover, our study showed that acyclovir was able to influence ALDH activity in the breast cancer cells. The ALDH superfamily consists of 19 isoenzymes with various cellular localizations, tissue/organ distributions and functions. ALDH enzymes catalyze highly reactive aldehydes and some isoenzymes play structural roles related to the osmoregulation and possess antioxidant functions [30]. Following cancer stem cell theories, where cancer is suggested to have a stem origin, ALDH was found to be a common marker for both normal and cancer stem cells [22, 33, 34]. Increased level of ALDH enzyme is an indicator of a high tumorigenic potential of the cancer cell and ability to self-renew and initiate tumor progression [21]. In the breast cancer cells upregulated expression of ALDH is associated with a poor clinical outcome [19, 20]. Our results suggest that ACV affects multiple aspects of cellular life related to the carcinogenesis and ALDH fulfills the role of a marker for these changes.

A study by Curiel et al. reported that ACV had an inhibitory effect on one of the immune system components as T-regulatory cells (Treg) in glioblastomas through the suppression of indoleamine 2, 3-dioxygenase activity [35]. Another antiviral agent - ribavirin was also reported as an immune response inducer in the renal cell carcinoma lines through the downregulation of IL-10 expression and the upregulation of TGF-β expression [14]. The mechanisms by which ACV enables its anticancer effects might involve an enhanced immune response of the cancer cells and further study is required in this area.

There are several limitations in this study. All experiments were performed in vitro only on one cell line. Future research should focus on an adjuvant strategy of different antiviral agents to determine whether a combinatorial effect exists, and if so, which pathways are affected during the mechanism. Additionally, an examination of epigenetic modifications might serve as a platform for understanding the molecular mechanism underlying the antiviral therapy.

## Conclusion

In summary, we present evidence that ACV has an anticancer effect on breast cancer cell line. Our study shows that ACV was able to inhibit cancer cells proliferation, colony formation ability and cell invasion capacity, while having no effect on the secretion of certain tumor suppressor genes. Treatment with ACV induced downregulation of ALDH activity, suggesting a decrease of the tumorigenic potential of the treated cancer cells. These results provide new insights on the effect of antiviral agents on the tumorigenesis and metastasis. However, more research is necessary to identify the primary target of ACV and maximize its potential.

## Additional files

Additional file 1: Viability of MCF7 breast cancer and normal breast epithelial cells in response to acyclovir. Error bars represent 95% confidence interval based on the standard deviation. (*) indicates *p* <0.05 as compared with other samples and for pairwise comparison. One way ANOVA followed by Tukey’s test were used for statistical analysis. The data for each cell type were taken from same culture experiment. (DOCX 144 kb)

Additional file 2: Population doubling time (hours) of proliferation of MCF7 cells treated with ACV. Error bars represent 95% confidence interval based on the standard deviation. (*) indicates *p* <0.05 as compared with other samples and for pairwise comparison. One way ANOVA followed by Tukey’s test were used for statistical analysis. The data for each cell type were taken from same culture experiment. (DOCX 301 kb)

Additional file 3: Annexin V staining of apoptotic MCF7 cells after treatment with acyclovir. Left panel is early apoptosis, right panel is late apoptosis. Error bars represent 95% confidence interval based on the standard deviation. One way ANOVA followed by Tukey’s test were used for statistical analysis. Means are not significant, *p* > 0.05. *P*-value for early apoptosis = 1.31579; for late apoptosis = 0.91371. The data for each cell type were taken from same culture experiment. (DOCX 288 kb)

Additional file 4: Quantitative analysis of nucleus and cytoplasm of MCF7 breast cancer cells without and with acyclovir treatment. (DOCX 10 kb)

Additional file 5: A scatter plot of measurements where best fit line and a slope indicate rate of migrating cells. (DOCX 62 kb)

Additional file 6: NF-kB p65 (pg/10^3 cells) expression of MCF7 cells in response to ACV treatment. Error bars represent 95% confidence interval based on the standard deviation. (*) indicates *p* <0.05 as compared with other samples and for pairwise comparison. One way ANOVA followed by Tukey’s test were used for statistical analysis. The data for each cell type were taken from the same culture experiment. (DOCX 28 kb)

## Abbreviations

ACV: Acyclovir
ALDH: aldehyde dehydrogenase
BCS: Bovine calf serum
BSA: Bovine serum albumin
CMV: Cytomegalovirus
DMEM: Dulbecco’s modified eagle’s medium
DMSO: Dimethyl sulfoxide
EBV: Epstein–barr virus
eIF4E: Eukaryotic translation initiation factor 4E
ELISA: Enzyme-linked immunosorbent assay
EMT: Epithelial-to-mesenchymal transition
FF: Form factor
HBV: Hepatitis B
HCC: Hepatocellular carcinoma
HCV: Hepatitis C
hENT1: Human equilibrative nucleoside transporter 1
HPV: Human papillomavirus
HSV: Herpes simplex virus
IF: Immunofluorescence
MTT: 3-(4,5-dimethylthiazol-2- yl)-2,5-diphenyltetrazolium bromide
NAD: Nicotinamide adenine dinucleotide
NF-kB: Nuclear factor kappa-light-chain-enhancer of activated B cells
PBS: Phosphate buffered saline
PMSF: Phenylmethylsulfonyl fluoride
RIPA buffer: Radioimmunoprecipitation assay buffer
Treg: T-regulatory cells
