# Supplementary figures and images for: The effects of antiviral treatment on breast cancer cell line
Source: Infect Agent Cancer. 2017 Mar 23;12:18. doi: 10.1186/s13027-017-0128-7 (PMC5364572; doi:10.1186/s13027-017-0128-7)

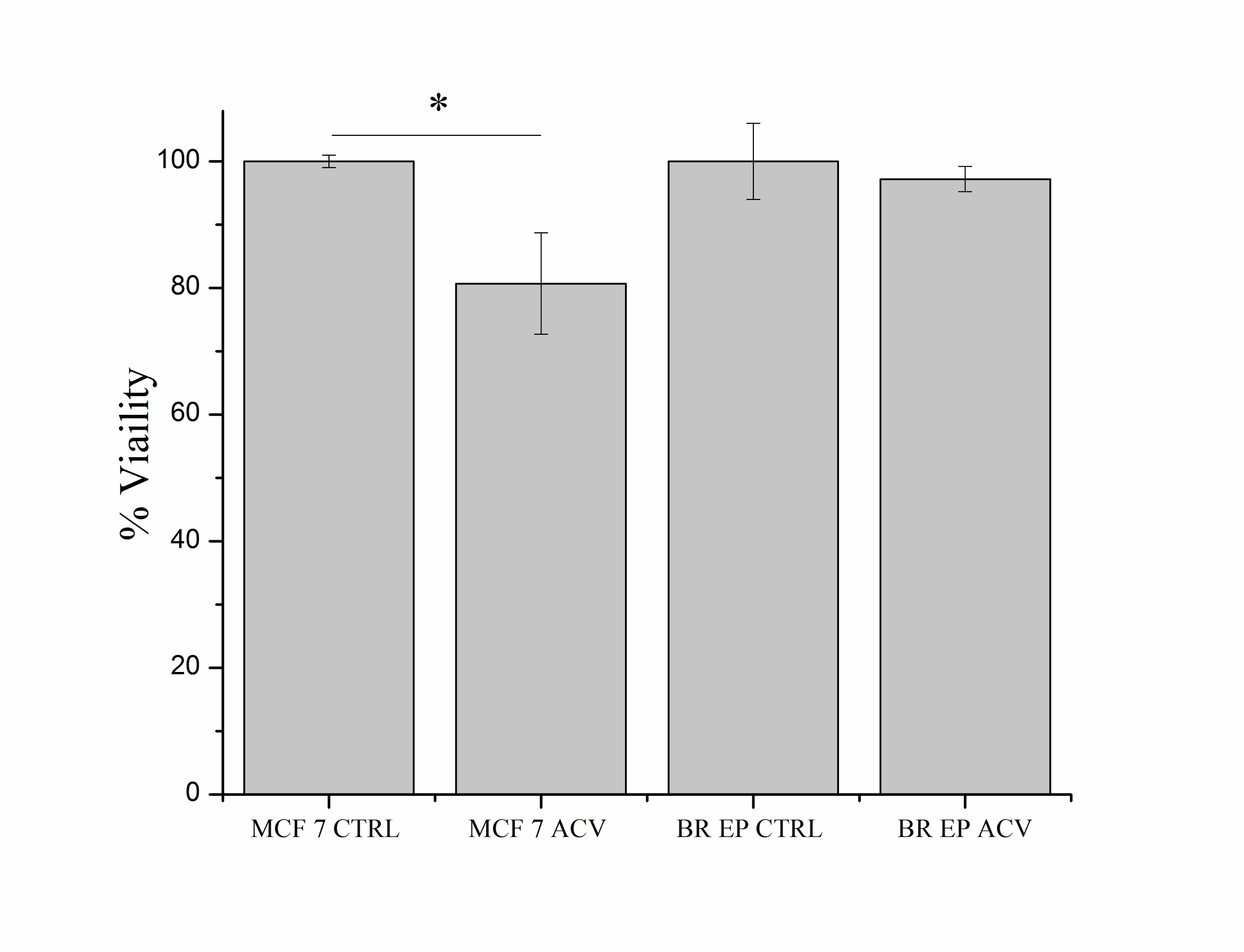

Supplement: Supplementary file 1 — Viability of MCF7 breast cancer and normal breast epithelial cells in response to acyclovir. Error bars represent 95% confidence interval based on the standard deviation. (*) indicates p <0.05 as compared with other samples and for pairwise comparison. One way ANOVA followed by Tukey’s test were used for statistical analysis. The data for each cell type were taken from same culture experiment. (DOCX 144 kb) [file 13027_2017_128_MOESM1_ESM.docx]

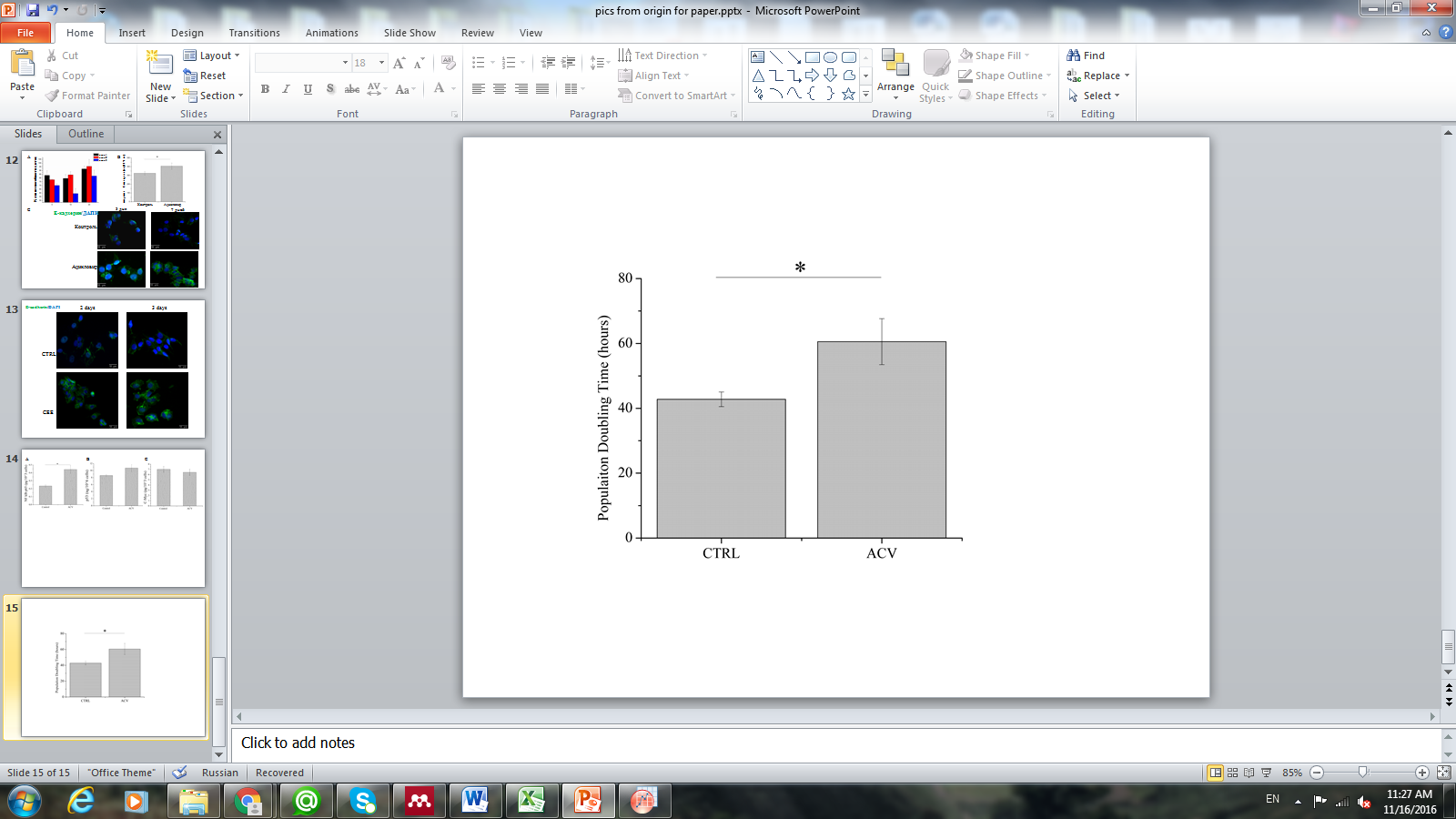

Supplement: Supplementary file 2 — Population doubling time (hours) of proliferation of MCF7 cells treated with ACV. Error bars represent 95% confidence interval based on the standard deviation. (*) indicates p <0.05 as compared with other samples and for pairwise comparison. One way ANOVA followed by Tukey’s test were used for statistical analysis. The data for each cell type were taken from same culture experiment. (DOCX 301 kb) [file 13027_2017_128_MOESM2_ESM.docx]

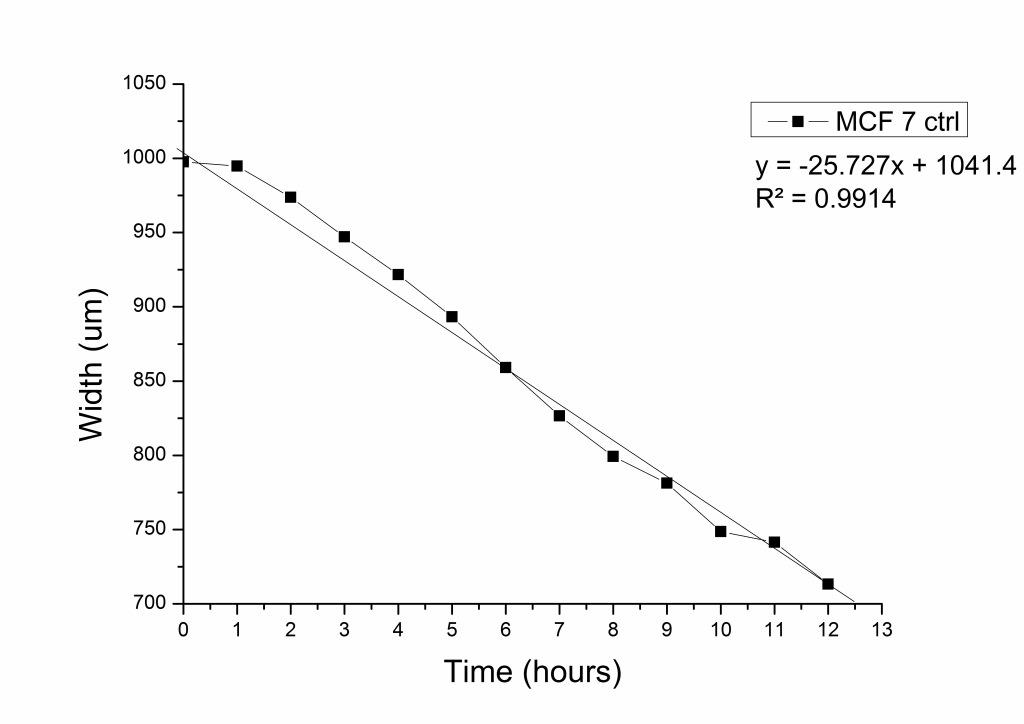

Supplement: Supplementary file 5 — A scatter plot of measurements where best fit line and a slope indicate rate of migrating cells. (DOCX 62 kb) [file 13027_2017_128_MOESM5_ESM.docx]
