# Supplementary material for: The effects of antiviral treatment on breast cancer cell line
Source: Infect Agent Cancer. 2017 Mar 23;12:18. doi: 10.1186/s13027-017-0128-7 (PMC5364572; doi:10.1186/s13027-017-0128-7)
Supplement: Supplementary file 3 — Annexin V staining of apoptotic MCF7 cells after treatment with acyclovir. Left panel is early apoptosis, right panel is late apoptosis. Error bars represent 95% confidence interval based on the standard deviation. One way ANOVA followed by Tukey’s test were used for statistical analysis. Means are not significant, p > 0.05. P-value for early apoptosis = 1.31579; for late apoptosis = 0.91371. The data for each cell type were taken from same culture experiment. (DOCX 288 kb) [file 13027_2017_128_MOESM3_ESM.docx]

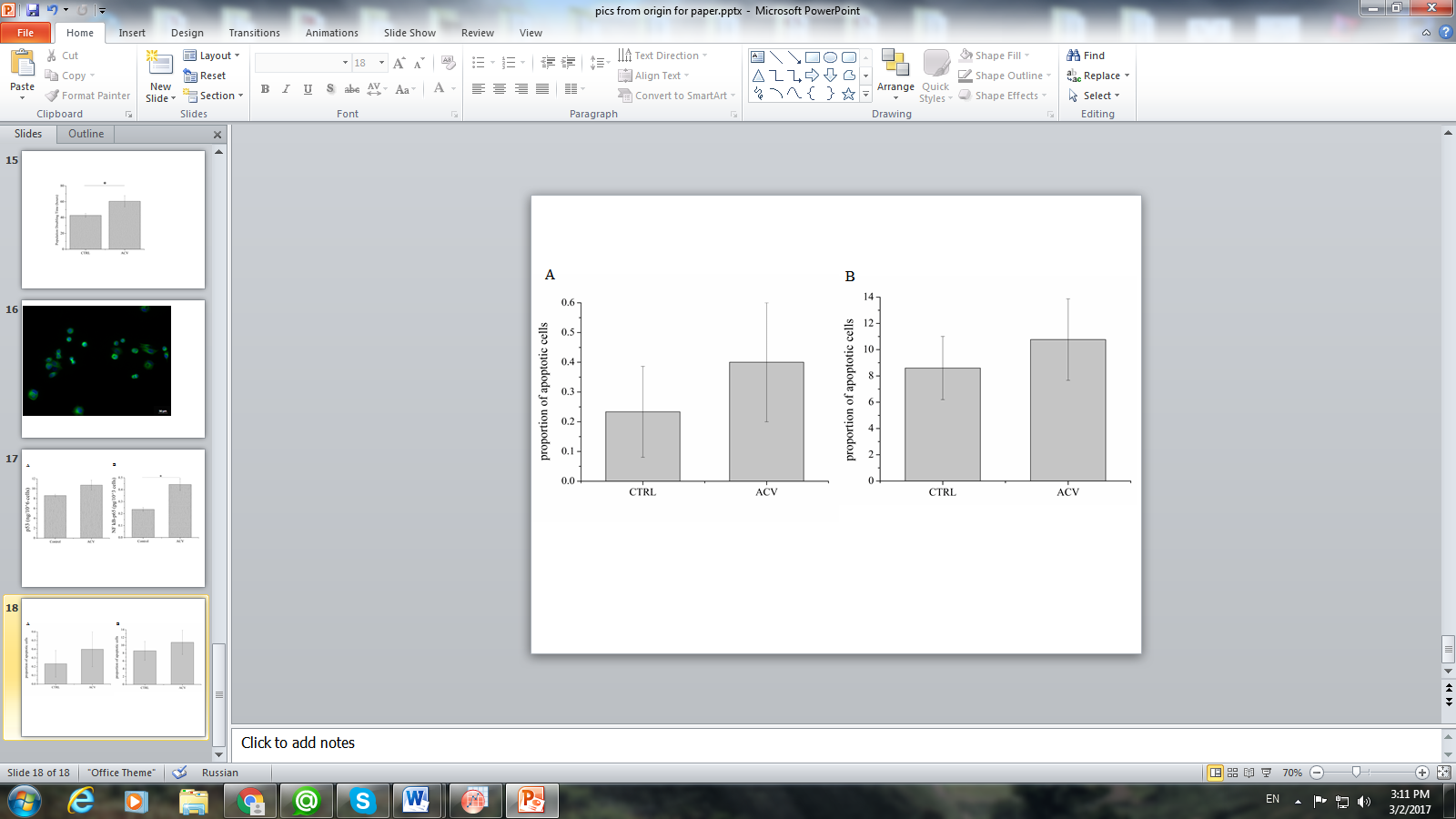


1. Early apoptosis. Means are not significant, p-value>0.05. P-value = 1.31579.
2. Late apoptosis. Means are not significant, p-value>0.05. P-value = 0.91371.
