# Supplementary material for: The effects of antiviral treatment on breast cancer cell line
Source: Infect Agent Cancer. 2017 Mar 23;12:18. doi: 10.1186/s13027-017-0128-7 (PMC5364572; doi:10.1186/s13027-017-0128-7)
Supplement: Supplementary file 4 — Quantitative analysis of nucleus and cytoplasm of MCF7 breast cancer cells without and with acyclovir treatment. (DOCX 10 kb) [file 13027_2017_128_MOESM4_ESM.docx]

Additional file 4. Quantitative analysis of nucleus and cytoplasm of MCF7 breast cancer cells without and with acyclovir treatment

|  | Untreated MCF7 cells | | ACV treated MCF7 cells | |
| --- | --- | --- | --- | --- |
|  | Nucleus | Cytoplasm | Nucleus | Cytoplasm |
| FF | 0.869±0.014 | 0.828±0.014 | 0.881±0.014 | 0.659±0.012 |
| Area ratio (nucleus/  cytoplasm) | 0.730 | | 0.810 | |
