# Supplementary material for: The effects of antiviral treatment on breast cancer cell line
Source: Infect Agent Cancer. 2017 Mar 23;12:18. doi: 10.1186/s13027-017-0128-7 (PMC5364572; doi:10.1186/s13027-017-0128-7)
Supplement: Supplementary file 4 [file 13027_2017_128_MOESM6_ESM.docx]

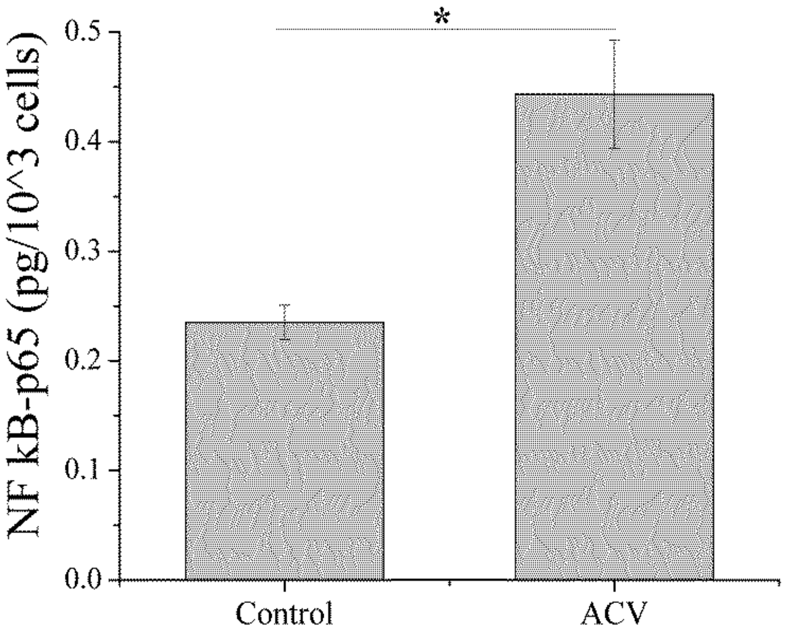

*
NF kB-p65 (pg/10^3 cells)
0.5
0.4
0.3
0.2
0.1
0.0
Control
ACV
